# The Editor's Letter-Box

**Published:** 1913-08-02

**Authors:** J. Roberson Day

**Affiliations:** 31 Devonshire Place, W.


### THE EDITOR'S LETTER-BOX.

### Saline Injections in Infantile Diarrhœa.

*To the Editor of* The Hospital.

*To the Editor of* The Hospital.

Sir,—With further reference to this subject, your footnote to my letter which you publish in to-day's issue shows clearly that you have overlooked a very important point in the Plasma de Quinton. The article in the *Practitioner* for July I only saw *after* writing my letter. I did not "discreetly ignore" it, but I now refer to it.

Professor H. B. Day evidently did not make use of Quinton's marine plasma, although he thinks he did, and you, Sir, have fallen into the same error. He says the sea-water was diluted with sterilised distilled water and the whole heated in an autoclare to ensure sterility. This was done, he tells us, on account of the cost of the Plasma de Quinton as supplied from Paris.

Now M. Quinton emphasises the necessity of diluting the pure sea-water with a pure spring-water as free as possible from mineral constituents; with this spring-water the sea-water is made isotonic, and the mixture is filtered through a Pasteur-Chamberland filter and put into leadless glass ampoules but not sterilised by heat, and no antiseptic is added. Herein lies the difference, but this makes all the difference, and we cannot accept the results Professor Day arrives at, interesting as they would have been, because he has not been working with Quinton's plasma, although he apparently thinks he has.

With his greater opportunities for testing the relative advantages of the various saline injections now employed, I for one hope he will be induced to repeat his observations, but he must use modified sea-water as directed by Quinton. We know that infants fed exclusively on sterilised milk are liable to develop scurvy, because an intangible something has been destroyed by heating. Is it not possible that something of the same kind happens when water is boiled?

I should like to see the back numbers of The Hospital to which you refer; kindly send them to me.—Yours faithfully,

J. Roberson Day, M.D.(Lond.).

31 Devonshire Place, W.,
July 26, 1913.

[Our reply to Dr. Burford's letter (see previous page) will show that Dr. J. R. Day is wrong in supposing that Professor Day has not used the Quinton fluid. He has, on the contrary, tried it and compared it with similar fluid made as Dr. J. R. Day describes. The fact that he can find no difference between the two fluids knocks the bottom out of Dr. J. R. Day's argument that there is some subtle difference between them.—Ed. The Hospital.]

